# Effects of an Immersive Virtual Reality Exergame on University Students’ Anxiety, Depression, and Perceived Stress: Pilot Feasibility and Usability Study

**DOI:** 10.2196/29330

**Published:** 2021-11-22

**Authors:** Wenge Xu, Hai-Ning Liang, Nilufar Baghaei, Xiaoyue Ma, Kangyou Yu, Xuanru Meng, Shaoyue Wen

**Affiliations:** 1 Digital Media Technology Lab School of Computing and Digital Technology Birmingham City University Birmingham United Kingdom; 2 Department of Computing School of Advanced Technology Xi'an Jiaotong-Liverpool University Suzhou China; 3 School of Natural and Computational Sciences Massey University Auckland New Zealand; 4 School of Information Technology and Electrical Engineering University of Queensland Brisbane Australia

**Keywords:** university students, depression, anxiety, stress, immersive virtual reality, exergame

## Abstract

**Background:**

In recent years, there has been an increase in the number of students with depression, anxiety, and perceived stress. A solution that has been increasingly used for improving health and well-being is exergaming. The effects and acceptability of exergames have been studied widely but mostly with older adults. The feasibility and usability of exergames among university students, especially those of immersive virtual reality (iVR) exergames, remain unexplored.

**Objective:**

This study aimed to explore the feasibility of a 6-week iVR exergame–based intervention in reducing anxiety, depression, and perceived stress among university students and to examine the usability and acceptability of such games.

**Methods:**

A total of 31 university students were recruited to participate in a 6-week study in which they needed to play a boxing-style iVR exergame called *FitXR* (FitXR Limited) twice per week (30 minutes per session). Their anxiety (Beck Anxiety Inventory), depression (Beck Depression Inventory-II), and perceived stress (Perceived Stress Scale) levels were measured before and after intervention.

**Results:**

A total of 15 participants completed the 6-week study. Our results suggested that participants’ mean depression scores decreased significantly from 8.33 (SD 5.98) to 5.40 (SD 5.14) after the intervention (*P*=.01). In addition, most participants (14/15, 93%) believed that the iVR exergame has good usability. Furthermore, most participants (14/15, 93%) were satisfied with the iVR gameplay experience and would play the iVR exergame again in the future. Of the 15 participants, 11 (73%) would recommend the iVR exergame to their friends.

**Conclusions:**

The results gained from this study show that the iVR exergame has good usability, is highly acceptable, and has the potential to reduce depression levels among university students.

## Introduction

### Background

There is growing evidence that the number of university students who are struggling with mental health problems is increasing globally (eg, in North America [1], Japan [2], and the United Kingdom [3]). Among these mental health problems, university students are particularly at high risk for anxiety, depression, and stress [2,4-9]. These mental health problems can result in different consequences among students, such as university dropout, decreased academic performance and social functioning, and even suicidal behavior [10,11].

One solution that is widely accepted is to provide support via counseling centers located within university campuses. Consistent with the rising rates of depression and anxiety, the number of students seeking mental health services in campus counseling centers has increased. Research has shown that the percentage of students who have received treatment at a university counseling center has increased from 6.6% in 2007 to 11.8% in 2017 [12]. Consequently, these counseling centers often report that they are over capacity and are unable to immediately meet the needs of the large number of students who are requesting services. This is because counseling centers have limitations such as a limited number of available sessions, long waiting lists, and a limited amount of staff [13], which can result in unwanted outcomes for students with mental health problems.

Sports-based interventions are useful for achieving health benefits—both mental and physical benefits—and provide more flexibility and ease of access for patients than those provided by counseling centers. Exercises involving large muscle groups in the whole body, especially those that involve following rhythmic flow patterns, can effectively alleviate depression [14]. A typical example of this type of exercise is boxing. Using boxing as a form of therapy can help with stress and anger management, boost confidence and self-esteem, elevate mood, serve as a natural antianxiety activity, and improve focus and sleep quality [15]. Boxing training exercises may have a better therapeutic effect on obesity, cardiovascular, and health-related quality of life outcomes than an equivalent dose of brisk walking [16]. In addition, boxing may be the most beneficial to those who are going through the early to middle stages of Parkinson disease progression [17]. For instance, therapeutic boxing can positively affect the speech, social interaction skills, and mental health of individuals with Parkinson disease [18]. It can also improve older adults’ gait, balance, daily living activities, and quality of life [19]. Further, one study suggests that shadow boxing (ie, the practice of committing repetitive boxing movements to muscle memory), together with psychosomatic relaxation, has a beneficial auxiliary therapeutic effect on depression and anxiety among people with type 2 diabetes [20].

In recent years, exergaming, which combines video games and physical exercises, has been widely used to promote both physical and mental health [21,22] in different population groups (ie, children [23], young individuals [24], and older adults [25]). However, most studies have been conducted with non–immersive virtual reality (iVR) exergames [26] (ie, those played on flat-screen televisions or computer monitors) [27-29]. On the other hand, the use of iVR exergame–based interventions is still underexplored.

iVR exergames [30-32] have been gaining attention rapidly due to the recent emergence of affordable iVR head-mounted displays. iVR exergames have many advantages over non-iVR exergames; they can provide more positive game experiences to players compared to those provided by non-iVR exergames [32,33]. Furthermore, exercising within iVR can result in higher increases in enjoyment and motivation compared to those resulting from playing exergames with standard televisions or computer monitors [34]. As such, exergaming in iVR might increase people’s adherence to regular physical exercise in general [35-37].

### Goal of This Study

The goal of this study was to evaluate the usability and acceptability of an iVR exergame (FitXR; FitXR Limited) [38] for university students. We also wanted to examine the feasibility and usability of the iVR exergame by conducting a 6-week pilot study on reducing anxiety, depression, and perceived stress levels among university students.

## Methods

### Recruitment

Students were recruited from a local university campus through physical and digital advertisements (ie, posters, social media platforms, and a mailing list). The inclusion criteria were as follows: (1) enrolled as a full-time student, (2) aged at least 18 years, and (3) was not pregnant (because of the physical exertion required to play the game). The exclusion criterion was a “yes” answer in the Physical Activity Readiness Questionnaire [39].

### Intervention

**Figure 1:**
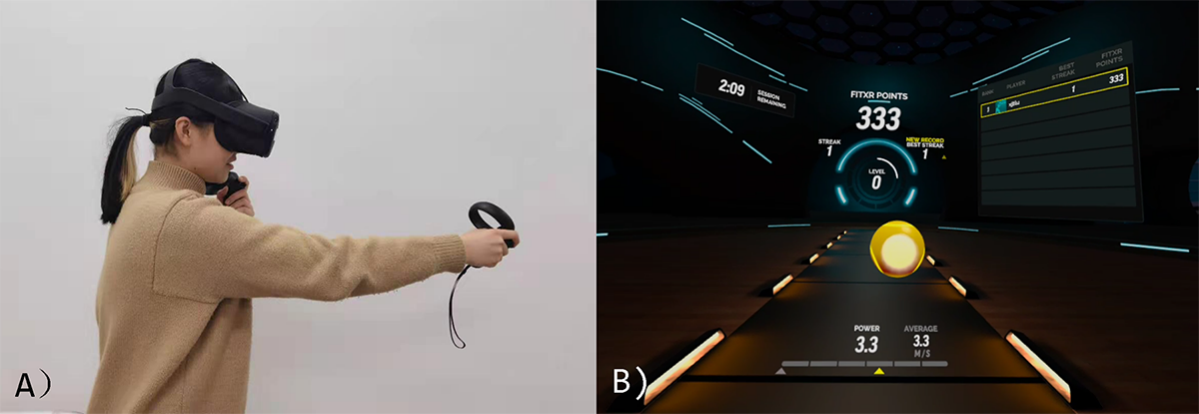
(A) An example of a participant playing the immersive virtual reality exergame by using the Oculus Quest 1 (Oculus). (B) A screenshot of the exergame.

Eligible participants were invited to an indoor laboratory room that could not be seen from the outside. Participants first completed an in-person consent form. Then, they needed to fill in the preexperiment questionnaire, which was used to collect their demographic information and anxiety, depression, and perceived stress baselines (see *Outcome Measures* section for details on the questionnaires used in the experiment). An experimenter helped participants put on an Oculus Quest 1 (Oculus) when they completed the preexperiment questionnaire and instructed them on how to play the FitXR exergame (Figure 1). Once they were familiar with the device and the game, they started their first training session.

Eligible participants were scheduled for the iVR exergame intervention twice per week (not on the same day) for 6 weeks. Each session consisted of about 30 minutes of gameplay. The game provided 11 game levels that ranged in duration from 26 minutes to 31 minutes. We selected the game levels that had around 30 minutes of gameplay. Unlimited drinks and snacks were provided during each session. At the end of the last session, participants were asked to fill out the postexperiment questionnaire, which was used to measure participants’ anxiety, depression, and perceived stress levels and the acceptability and usability of the game.

FitXR was selected because (1) it is a boxing-inspired iVR fitness game; (2) it involves a great number of jabs, uppercuts, defensive actions (ie, cover-ups), squats, and leftward and rightward movements; (3) it involves few rotational movements, which is helpful for potentially avoiding motion sickness [40]; and (4) it provides several unique training sessions from which players can choose. To play the game successfully, players need to perform several punching combos (jab or uppercut) on sphere objects and avoid getting hit by blocks by performing squats and lunges.

### Outcome Measures

#### Anxiety

The Beck Anxiety Inventory (BAI) [41] is a 21-item self-report scale that ranges from 0 to 63, with higher scores indicating more severe anxiety. The BAI has achieved a Cronbach α of .92, thereby demonstrating its internal consistency [41,42]. A review paper on anxiety questionnaires indicated that the BAI is suitable for the general population, has good reliability, and has moderate validity [43]. Further, the BAI has been used in studies dealing with exercise [44-46] and in exergame studies [22].

#### Depression

We measured depression levels by using the Beck Depression Inventory-II (BDI-II) [47], which consists of a 21-item self-report scale that ranges from 0 to 63, with higher scores indicating more severe depression. This scale has a Cronbach α coefficient of .92 and a test-retest reliability value of 0.93. A review paper on depression questionnaires indicated that the BDI-II is suitable for the general population, and it has excellent reliability and good validity [48]. The BDI-II has been used in studies dealing with exercise [44,45,49].

#### Perceived Stress

Perceived stress levels were measured via the widely used Perceived Stress Scale [50], which consists of 14 items questions with scores that range from 0 (never) to 4 (very often). This questionnaire has been used in studies dealing with exercise [45,51,52] and with exergame-based interventions [53,54].

#### Usability and Acceptability

Usability and acceptability were only tested at the end of the last session via self-reported questionnaires. Usability was measured by using the System Usability Scale (SUS). SUS scores range between 0 and 100; 100 represents the best usability, and a score of ≥68 is considered positive [55].

We measured acceptability and satisfaction with the following items, which were answered by using a 5-point Likert scale (1 indicated “extremely disagree” and 5 indicated “extremely agree”): “I was satisfied with FitXR experience,” “I would play FitXR again in the future,” and “I would recommend FitXR to a friend.” Finally, we asked the participants the following questions: “How often would you play FitXR per week” and “How long would you play FitXR each day?”

### Statistical Power and Analysis

Analyses were performed by using SPSS version 24.0 (IBM Corporation). The normality of the data was tested by using Shapiro-Wilk tests. We used 2-tailed paired *t* tests, in which time (week 1: pretest; week 6: posttest) was used as the within-subjects variable, if the differences in the dependent variable between pretest and posttest times were normally distributed. Otherwise, the Wilcoxon signed-ranks test was used.

## Results

### Participants’ Characteristics

A total of 31 participants volunteered to take part in this study, and in the end, 15 completed it. The reasons for dropout included urgent family reasons (n=1); health-related reasons, such as discomfort due to menstrual periods (n=2); high coursework workloads (n=2); complaints that the selected game was not as good as expected (n=2); the belief that the exertion level of the game was too high (n=2); and physical reasons (n=1). Further, 6 people did not provide a reason for dropping out. Table 1 shows the demographics of the participants and their experience with iVR and exergames.

**Table 1:** Characteristics of the study participants.

Characteristic		Value
Students, n (number of males)		15 (8)
Age (years), mean (SD)		19.1 (0.96)
BMI, mean (SD)		21.7 (2.98)
**Participants who had self-reported experience with exergames, n**		7
	Frequent user (daily or weekly)	1
**Participants who had self-reported experience with iVR^a^ HMDs^b^, n**		7
	Frequent user (daily or weekly)	0

^a^iVR: immersive virtual reality.

^b^HMD: head-mounted display.

### Evaluation Outcomes

#### BAI—Anxiety

The mean anxiety score for the pretest was 4.87 (SD 3.98), and the mean anxiety score for the posttest was 5.40 (SD 5.41). The results of the 2-tailed paired samples *t* test showed that there was no significant difference between the pretest and posttest scores for anxiety (*t*_14_=−0.541; *P*=.60).

#### BDI-II—Depression

The mean depression score for the pretest was 8.33 (SD 5.98), and the mean depression score for the posttest was 5.40 (SD 5.14). The Wilcoxon signed-rank test indicated that the posttest depression scores were significantly lower than the pretest depression scores (*Z*=−2.526; *P*=.01) and that the intervention had a large effect on depression (effect size: *r*=−0.652). Overall, after playing the iVR boxing exergame for 6 weeks, the university students in this study reported being less depressed.

#### Perceived Stress

The mean perceived stress score for the pretest was 16.87 (SD 4.88), and the mean perceived stress score for the posttest was 16.13 (SD 5.81). A 2-tailed paired *t* test indicated that there was no significant difference between the pretest and posttest scores (*t*_14_=0.564; *P*=.58).

#### Usability and Acceptability

The SUS has a range of 0 to 100 and is used to rate the usability of an application. A score of ≥68 is deemed to be positive (ie, scores of 68: okay; scores of 68-80.3: good; scores of >80.3: excellent). The participants rated FitXR with a mean SUS score of 79.5 (SD 9.51); 7 participants rated it as excellent, another 7 rated it as good, and 1 rated it as poor. The highest SUS score was 97.5, while the lowest SUS score was 57.5.

With regard to the three satisfaction items, participants gave a mean score of 4.33 (SD 0.62) to the “I was satisfied with FitXR experience” item; 6 participants gave an “extremely agree” rating, 8 participants gave an “agree” rating, and 1 participant gave a “neutral” rating. As for the “I would play FitXR again in the future” item, participants gave an average score of 4.27 (SD 0.59); 5 participants gave an “extremely agree” rating, 9 participants gave an “agree” rating, and 1 participant gave a “neutral” rating. With regard to the “I would recommend FitXR to a friend” item, participants gave an average score of 4 (SD 9.26); 5 participants gave an “extremely agree” rating, 6 participants gave an “agree” rating, 3 participants gave a “neutral” rating, and 1 participant gave a “disagree” rating.

Lastly, participants indicated that they would like to play FitXR for an average of 3.87 days (SD 1.06 days) per week and would play the game for an average of 35 minutes (SD 25.93 minutes) each day.

## Discussion

### Principal Results

This study investigated the usability and acceptability of a boxing iVR exergame–based training intervention among university students and evaluated the intervention’s feasibility in a 6-week training program for reducing anxiety, depression, and perceived stress levels. The results of this pilot study show (1) that the FitXR iVR exergame was perceived to have good usability (ie, good usability scores) and was highly acceptable among university students, and (2) that playing the iVR boxing exergame for 6 weeks (ie, 2 sessions per week; 30 minutes per session) would likely reduce depression levels among university students.

### Comparison With Prior Work

There is evidence that exergames can significantly improve depression among people with depression (ie, among adults [56] and older adults [22,57,58]). The literature also suggests that exergames can improve depression in healthy subjects. For instance, depression among healthy older adults improved after playing Wii Fit (Nintendo) exergames twice per week for 4 weeks [59]; participants in the control group who participated in an education program for the same duration did not experience such improvements. A recent review also suggested that iVR therapies are effective in supporting the treatment of anxiety and depression [60].

Although university students are at high risk for mental issues [2,4-9], limited research has looked into using exergames to promote health among university students. A previous study suggested that playing a virtual reality exergame 3 times per week for 6 weeks could improve depression in healthy university students [61]. Our results also support this finding, as the depression scores measured by the BDI-II further decreased after participants played the iVR head-mounted display–based exergame for 6 weeks (pretest score: 8.33; posttest score: 5.40). However, we failed to obtain results that reflected whether playing an exergame could improve anxiety among healthy university students [61].

In terms of perceived stress, Huang et al [54] conducted a study to explore how playing exergames impacts the mood states (including perceived stress) of healthy university students and staff. They found that playing exergames for 30 consecutive minutes each week for 2 weeks could reduce perceived stress levels. Cutter et al [53] conducted an 8-week trial in which methadone-maintained patients played an exergame 5 times per week (session duration ranged from 20 to 25 minutes per session). They found that the exergame could reduce perceived stress levels. However, we could not obtain similar results for perceived stress.

Viana et al [62] found that playing an exergame (even for only 1 session) seemed to be a useful method for reducing state anxiety in healthy women. However, this is also not supported by our study on an iVR exergame.

With regard to usability, FitXR, in line with other exergames such as SliverFit (SUS score: mean 87.0) [63] and the rehabilitation exergame (SUS score: mean 89.6) developed by Uzor and Baillie [64], was rated with a good usability score (mean 79.5). This indicates that players can learn how to play and use the game quickly and that the game is very easy to use.

In line with a study by Yunus et al [61], we observed that university students were willing to accept playing an iVR-based exergame as a form of exercise. FitXR was perceived to be acceptable, and the majority of our participants (11/15, 73%) were glad to recommend FitXR to their friends.

The retention rate of our study was 48% (15/31). Furthermore, even though only about half of the participants (15/31, 48%) completed this study, the participants who dropped out mainly did so due to personal reasons (n=5), and only a few (n=2) complained that the game was not as good as expected. These data provide further insight into the potential of using the iVR exergame as an intervention for studying mental health among university students.

### Limitations and Future Work

There are 2 main limitations in this study. First, the lack of a control group did not allow for comparisons with people who undergo traditional therapy or do not undergo therapy at all. Since this pilot study has confirmed the feasibility of using the iVR exergame to reduce depression levels, it is worth conducting future studies on the impacts that iVR exergames have on health. Such studies could, for instance, use a more established study design, such as a randomized controlled trial involving people who undergo traditional therapy and a control group (people who do not undergo therapy). Second, no follow-up tests were conducted after the experiment. Therefore, it is unclear if and how long the observed benefits were maintained.

Like many other studies on usability and acceptability [65], we tested the exergame in a supervised environment. Future work could test the game in an unsupervised setting. In addition, we aim to develop our own iVR exergame prototype involving individualized and personalized features, such as avatars, environments, and behaviors, to improve gameplay engagement further [66,67].

### Conclusions

In this study, we investigated whether playing an iVR exergame for over 6 weeks could improve university students’ mental health (anxiety, depression, and perceived stress). We also explored the acceptability of the game and its usability. Our results indicated that the exergame has a good usability score and is highly acceptable among university students as a form of exercise. In addition, our findings showed that playing the exergame twice per week for 6 weeks can reduce depression scores among healthy university students.

## Abbreviations

BAI: Beck Anxiety Inventory
BDI-II: Beck Depression Inventory-II
iVR: immersive virtual reality
SUS: System Usability Scale
XJTLU: Xi’an Jiaotong-Liverpool University

## References

[1] LeViness P, Bershad C, Gorman K, Braun L, Murray T The Association for University and College Counseling Center Directors Annual Survey 2018. Association for University and College Counseling Center Directors.

[2] Tomoda A, Mori K, Kimura M, Takahashi T, Kitamura T (2000). One-year prevalence and incidence of depression among first-year university students in Japan: a preliminary study. Psychiatry Clin Neurosci.

[3] Singleton N, Bumpstead R, O'Brien M, Lee A, Meltzer H (2003). Psychiatric morbidity among adults living in private households, 2000. Int Rev Psychiatry.

[4] Nerdrum P, Rustøen T, Rønnestad MH (2007). Student psychological distress: A psychometric study of 1750 Norwegian 1st‐year undergraduate students. Scandinavian Journal of Educational Research.

[5] Ovuga E, Boardman J, Wasserman D (2006). Undergraduate student mental health at Makerere University, Uganda. World Psychiatry.

[6] Stewart-Brown S, Evans J, Patterson J, Petersen S, Doll H, Balding J, Regis D (2000). The health of students in institutes of higher education: an important and neglected public health problem?. J Public Health Med.

[7] Rith-Najarian LR, Boustani MM, Chorpita BF (2019). A systematic review of prevention programs targeting depression, anxiety, and stress in university students. J Affect Disord.

[8] Voelker R (2003). Mounting student depression taxing campus mental health services. JAMA.

[9] Wong JGWS, Cheung EPT, Chan KKC, Ma KKM, Tang SW (2006). Web-based survey of depression, anxiety and stress in first-year tertiary education students in Hong Kong. Aust N Z J Psychiatry.

[10] Keyes CLM, Eisenberg D, Perry GS, Dube SR, Kroenke K, Dhingra SS (2012). The relationship of level of positive mental health with current mental disorders in predicting suicidal behavior and academic impairment in college students. J Am Coll Health.

[11] Salzer MS (2012). A comparative study of campus experiences of college students with mental illnesses versus a general college sample. J Am Coll Health.

[12] Lipson SK, Lattie EG, Eisenberg D (2019). Increased rates of mental health service utilization by U.S. college students: 10-year population-level trends (2007-2017). Psychiatr Serv.

[13] Lattie E, Cohen KA, Winquist N, Mohr DC (2020). Examining an app-based mental health self-care program, IntelliCare for college students: Single-arm pilot study. JMIR Ment Health.

[14] Steptoe A, Bolton J (1988). The short-term influence of high and low intensity physical exercise on mood. Psychol Health.

[15] Shosha M (2020). A brief introduction to therapeutic boxing. International Journal of Physiology, Nutrition and Physical Education.

[16] Cheema BS, Davies TB, Stewart M, Papalia S, Atlantis E (2015). The feasibility and effectiveness of high-intensity boxing training versus moderate-intensity brisk walking in adults with abdominal obesity: a pilot study. BMC Sports Sci Med Rehabil.

[17] Morris ME, Ellis TD, Jazayeri D, Heng H, Thomson A, Balasundaram AP, Slade SC (2019). Boxing for Parkinson's disease: Has implementation accelerated beyond current evidence?. Front Neurol.

[18] Meinert R, Hatkevich B (2019). The effect of community-based therapeutic boxing on the speech, social interaction skills, and mental health of individuals with Parkinson’s disease. Am J Occup Ther.

[19] Long T, Robertson T (2007). Foundations of Therapeutic Recreation.

[20] Zheng Y, Zhou Y, Lai Q (2015). Effects of twenty-four move shadow boxing combined with psychosomatic relaxation on depression and anxiety in patients with type-2 diabetes. Psychiatr Danub.

[21] Pluchino A, Lee SY, Asfour S, Roos BA, Signorile JF (2012). Pilot study comparing changes in postural control after training using a video game balance board program and 2 standard activity-based balance intervention programs. Arch Phys Med Rehabil.

[22] Rosenberg D, Depp CA, Vahia IV, Reichstadt J, Palmer BW, Kerr J, Norman G, Jeste DV (2010). Exergames for subsyndromal depression in older adults: a pilot study of a novel intervention. Am J Geriatr Psychiatry.

[23] Hernandez HA, Ye Z, Graham TCN, Fehlings D, Switzer L (2013). Designing action-based exergames for children with cerebral palsy.

[24] Street TD, Lacey SJ, Langdon RR (2017). Gaming your way to health: A systematic review of exergaming programs to increase health and exercise behaviors in adults. Games Health J.

[25] Xu W, Liang HN, Baghaei N, Berberich BW, Yue Y (2020). Health benefits of digital videogames for the aging population: A systematic review. Games Health J.

[26] Zeng N, Pope Z, Lee JE, Gao Z (2018). Virtual reality exercise for anxiety and depression: A preliminary review of current research in an emerging field. J Clin Med.

[27] Huang HC, Nguyen HV, Cheng TCE, Wong MK, Chiu HY, Yang YH, Teng CI (2019). A randomized controlled trial on the role of enthusiasm about exergames: Players' perceptions of exercise. Games Health J.

[28] da Silva Alves R, Iunes DH, de Carvalho JM, Menezes FDS, Silva AM, Borges JBC, Carvalho LC (2018). Effects of exergaming on quality of life in cancer patients. Games Health J.

[29] Gao Y, Mandryk RL (2011). GrabApple: The design of a casual exergame. Entertainment Computing – ICEC 2011.

[30] Xu W, Liang HN, Yu Y, Monteiro D, Hasan K, Fleming C (2019). Assessing the effects of a full-body motion-based exergame in virtual reality.

[31] Xu W, Liang HN, He Q, Li X, Yu K, Chen Y (2020). Results and guidelines from a repeated-measures design experiment comparing standing and seated full-body gesture-based immersive virtual reality exergames: Within-subjects evaluation. JMIR Serious Games.

[32] Xu W, Liang HN, Yu K, Baghaei N (2021). Effect of gameplay uncertainty, display type, and age on virtual reality exergames.

[33] Xu W, Liang HN, Zhang Z, Baghaei N (2020). Studying the effect of display type and viewing perspective on user experience in virtual reality exergames. Games Health J.

[34] Farrow M, Lutteroth C, Rouse PC, Bilzon JLJ (2019). Virtual-reality exergaming improves performance during high-intensity interval training. Eur J Sport Sci.

[35] Frederick CM, Ryan RM (1993). Differences in motivation for sport and exercise and their relations with participation and mental health. J Sport Behav.

[36] Ryan RM, Frederick CM, Lepes D, Rubio N, Sheldon KM (1997). Intrinsic motivation and exercise adherence. Int J Sport Psychol.

[37] Alexandris K, Tsorbatzoudis C, Grouios G (2017). Perceived constraints on recreational sport participation: Investigating their relationship with intrinsic motivation, extrinsic motivation and amotivation. J Leis Res.

[38] FitXR - Exercise you'll love. FitXR Limited.

[39] Thomas S, Reading J, Shephard RJ (1992). Revision of the physical activity readiness questionnaire (PAR-Q). Can J Sport Sci.

[40] Keshavarz B, Hecht H (2011). Axis rotation and visually induced motion sickness: the role of combined roll, pitch, and yaw motion. Aviat Space Environ Med.

[41] Beck AT, Epstein N, Brown G, Steer RA (1988). An inventory for measuring clinical anxiety: psychometric properties. J Consult Clin Psychol.

[42] Halfaker DA, Akeson ST, Hathcock DR, Mattson C, Wunderlich TL, Lennard TA, Walkowski S, Singla AK, Vivian DG (2011). 3 - Psychological aspects of pain. Pain Procedures in Clinical Practice (Third Edition).

[43] Julian LJ (2011). Measures of anxiety: State-Trait Anxiety Inventory (STAI), Beck Anxiety Inventory (BAI), and Hospital Anxiety and Depression Scale-Anxiety (HADS-A). Arthritis Care Res (Hoboken).

[44] Ólafsdóttir KB, Kristjánsdóttir H, Saavedra JM (2018). Effects of exercise on depression and anxiety. A comparison to transdiagnostic cognitive behavioral therapy. Community Ment Health J.

[45] Paolucci EM, Loukov D, Bowdish DME, Heisz JJ (2018). Exercise reduces depression and inflammation but intensity matters. Biol Psychol.

[46] Rawson RA, Chudzynski J, Gonzales R, Mooney L, Dickerson D, Ang A, Dolezal B, Cooper CB (2015). The impact of exercise on depression and anxiety symptoms among abstinent methamphetamine-dependent individuals in a residential treatment setting. J Subst Abuse Treat.

[47] Beck AT, Steer RA, Brown GK (1996). Manual for the Beck Depression Inventory-II.

[48] Smarr KL, Keefer AL (2011). Measures of depression and depressive symptoms: Beck Depression Inventory-II (BDI-II), Center for Epidemiologic Studies Depression Scale (CES-D), Geriatric Depression Scale (GDS), Hospital Anxiety and Depression Scale (HADS), and Patient Health Questionnaire-9 (PHQ-9). Arthritis Care Res (Hoboken).

[49] Legrand FD (2014). Effects of exercise on physical self-concept, global self-esteem, and depression in women of low socioeconomic status with elevated depressive symptoms. J Sport Exerc Psychol.

[50] Cohen S, Kamarck T, Mermelstein R (1983). A global measure of perceived stress. J Health Soc Behav.

[51] Joshi AR, Pendse TN, Vaidya SM (2018). Effect of moderate aerobic exercise on perceived stress during luteal phase of menstrual cycle in students pursuing professional course. Natl J Physiol Pharm Pharmacol.

[52] Starkweather AR (2007). The effects of exercise on perceived stress and IL-6 levels among older adults. Biol Res Nurs.

[53] Cutter CJ, Schottenfeld RS, Moore BA, Ball SA, Beitel M, Savant JD, Stults-Kolehmainen MA, Doucette C, Barry DT (2014). A pilot trial of a videogame-based exercise program for methadone maintained patients. J Subst Abuse Treat.

[54] Huang HC, Wong MK, Yang YH, Chiu HY, Teng CI (2017). Impact of playing exergames on mood states: A randomized controlled trial. Cyberpsychol Behav Soc Netw.

[55] Brooke J, Jordan PW, Thomas B, McClelland IL, Weerdmeester B (1996). SUS: A 'quick and dirty' usability scale. Usability Evaluation In Industry.

[56] Yuen HK, Holthaus K, Kamen DL, Sword DO, Breland HL (2011). Using Wii Fit to reduce fatigue among African American women with systemic lupus erythematosus: a pilot study. Lupus.

[57] Li J, Theng YL, Foo S, Xu X (2018). Exergames vs. traditional exercise: investigating the influencing mechanism of platform effect on subthreshold depression among older adults. Aging Ment Health.

[58] Li J, Theng YL, Foo S (2016). Exergames for older adults with subthreshold depression: Does higher playfulness lead to better improvement in depression?. Games Health J.

[59] Chao YY, Scherer YK, Montgomery CA, Wu YW, Lucke KT (2015). Physical and psychosocial effects of Wii Fit exergames use in assisted living residents: a pilot study. Clin Nurs Res.

[60] Baghaei N, Chitale V, Hlasnik A, Stemmet L, Liang HN, Porter R (2021). Virtual reality for supporting the treatment of depression and anxiety: Scoping review. JMIR Ment Health.

[61] Yunus FW, Tan XZ, Romli MH (2020). Investigating the feasibility of exergame on sleep and emotion among university students. Games Health J.

[62] Viana RB, Alves CL, Vieira CA, Vancini RL, Campos MH, Gentil P, Andrade MS, de Lira CAB (2017). Anxiolytic effects of a single session of the exergame Zumba Fitness on healthy young women. Games Health J.

[63] Nawaz A, Skjæret N, Ystmark K, Helbostad JL, Vereijken B, Svanæs D (2014). Assessing seniors' user experience (UX) of exergames for balance training.

[64] Uzor S, Baillie L (2014). Investigating the long-term use of exergames in the home with elderly fallers.

[65] Nawaz A, Skjæret N, Helbostad JL, Vereijken B, Boulton E, Svanaes D (2016). Usability and acceptability of balance exergames in older adults: A scoping review. Health Informatics J.

[66] Baghaei N, Stemmet L, Khaliq I, Ahmadi A, Halim I, Liang HN, Xu W, Billinghurst M, Porter R (2021). Designing individualised virtual reality applications for supporting depression: A feasibility study.

[67] Baghaei N, Ahmadi A, Khaliq I, Liang HN (2021). Individualised virtual reality for supporting depression: Feedback from mental health professionals.

